# Molecular Mimicry of Transposable Elements in Plants

**DOI:** 10.1093/pcp/pcae058

**Published:** 2024-05-18

**Authors:** Jie Chu, Josephine Newman, Jungnam Cho

**Affiliations:** CAS Center for Excellence in Molecular Plant Sciences, Chinese Academy of Sciences, Shanghai, Beijing 200032, China; University of Chinese Academy of Science, Beijing 100049, China; Department of Biosciences, Durham University, Durham, DH1 3LE, UK; Department of Biosciences, Durham University, Durham, DH1 3LE, UK

**Keywords:** *Arabidopsis thaliana*, Extrachromosomal DNA, miRNA target mimic, *Oryza sativa*, Transcription factor sponge, Transposable elements

## Abstract

Transposable elements (TEs) are mobile DNA elements that are particularly abundant in the plant genomes. They have long been considered as junk DNA; however, a growing body of evidence suggests that TE insertions promote genetic diversity that is essential for the adaptive evolution of a species. Thus far, studies have mainly investigated the cis-acting regulatory roles of TEs generated by their insertions nearby or within the host genes. However, the trans-acting effects of TE-derived RNA and DNA remained obscure to date. TEs contain various regulatory elements within their sequences that can accommodate the binding of specific RNAs and proteins. Recently, it was suggested that some of these cellular regulators are shared between TEs and the host genes, and the competition for the common host factors underlies the fine-tuned developmental reprogramming. In this review, we will highlight and discuss the latest discoveries on the biological functions of plant TEs, with a particular focus on their competitive binding with specific developmental regulators.

## Introduction

Transposable elements (TEs) are DNA sequences that can move around the genome and are found in most organisms (Bourque et al. 2018). Although they are often deemed as ‘junk DNA’ (Palazzo et al. 2014), many studies have shown that TEs are fundamental for life, acting as a driver of evolution (Lisch 2013). TEs can be divided into two classes defined by their modes of transposition (Wicker et al. 2007). Class I TEs (or retrotransposons) mobilize through a ‘copy-and-paste’ mechanism where its RNA intermediates reverse transcribe to form a new element (Dombroski et al. 1994). Class II TEs (or DNA transposons) move by a ‘cut-and-paste’ mechanism where TEs are excised from their original location and reintegrated at a new position (Feschotte and Pritham 2007, Muñoz-López and García-Pérez 2010). In the plant genomes, the long terminal repeat (LTR) retrotransposon family is generally most widespread (Vitte et al. 2014). The propagation of LTR retrotransposons involves the conversion of mRNA into extrachromosomal DNA (ecDNA), which is mediated by the retrotransposon-encoded reverse transcriptase. Subsequently, the integrase transports ecDNA into the nucleus for genomic insertion (Sabot and Schulman 2006, Rebollo et al. 2012, Makarevitch et al. 2015, Griffiths et al. 2018, Cho et al. 2019).

TEs are normally repressed by epigenetic silencing pathways, which include histone and DNA methylation (Slotkin and Martienssen 2007). Histone H3 lysine 9 di-methylation (H3K9me2) and H3K27me1 are the major modifications present in constitutively silent TEs (Roudier et al. 2011, Zhang et al. 2018). DNA methylation of TEs is mediated via the RNA-directed DNA methylation (RdDM) pathway, in which the small interfering (si) RNA-Argonaute 4 (AGO4) complex recruits DNA methyltransferases to target loci (Matzke and Mosher 2014). Methylation of DNA is further maintained by METHYLTRANSFERASE 1 and CHROMOMETHYLASE 3 depending on the cytosine context (Zhang et al. 2018). In *Arabidopsis*, while most TEs are marked by DNA methylation (Ahmed et al. 2011), young TEs that have recently transposed display higher DNA methylation levels (Quadrana et al. 2016). Apart from chromatin-level repression, TEs are subject to post-transcriptional suppression. In the so-called alternative RdDM, RNA-DEPENDENT RNA POLYMERASE 6 acts on truncated TE transcripts (cleaved by the microRNAs or RNA surveillance pathways) and initiates the production of 21/22-nt siRNAs, which trigger RNA cleavage and further contribute to TE repression (Nuthikattu et al. 2013, De Tomás and Vicient 2023).

The impact of TEs on gene expression has mainly been investigated in the context of DNA sequence rearrangements (Ayarpadikannan and Kim 2014). For example, TEs can function as promoters by serving as a rich source of cis-regulatory elements that confer tissue- and/or stress-specific responsiveness (Peaston et al. 2004, Hirsch and Springer 2017, Batista et al. 2019, Uzunovic et al. 2019). An example is the pigment accumulation of citrus fruits, which is controlled by a MYB transcription factor that activates anthocyanin production. In blood oranges, for instance, a retrotransposon is inserted in the promoter region of this gene, triggering its activation and enhancing anthocyanin biogenesis (Butelli et al. 2012). Similar examples can be also found in apple and grapefruit, implying that TEs have played crucial roles in diversifying agricultural traits (Kobayashi et al. 2004, Telias et al. 2011).

TE insertion may affect nearby genes through altering their epigenetic status (Ahmed et al. 2011, Quadrana et al. 2016). For example, *FWA* is a strong repressor of flowering in *Arabidopsis* (Kinoshita et al. 2006, Fujimoto et al. 2008). Its promoter region contains a short interspersed nuclear element (SINE), and DNA methylation of this retrotransposon is required for the epigenetic repression of *FWA* (Soppe et al. 2000, Quesneville 2020). In addition, the *BONSAI* locus in *Arabidopsis* is flanked by a long interspersed nuclear element (LINE). In the mutant of *INCREASED BONSAI METHYLATION 1* (*IBM1*), which encodes a Jumonji-family histone demethylase, the increased H3K9me2 as well as DNA methylation at the LINE locus spreads toward the *BONSAI* locus, leading to its epigenetic silencing and unique bonsai phenotype (Saze and Kakutani 2007, Quesneville 2020).

In addition to the DNA level of gene expression control, TEs also play a role in post-transcriptional regulation of gene expression by providing alternative splicing and polyadenylation sites, thereby diversifying gene structure and function (Mayr 2017). Moreover, TEs can evolve to long non-coding RNAs (lncRNAs), which can interact with other RNAs and proteins to regulate gene expression (Kapusta and Feschotte 2014, Cho 2018). Overall, TEs can exert regulatory roles in various steps of gene expression, from DNA to RNA.

Although TEs are usually found transcriptionally inactive under normal conditions, some TEs can be reactivated by environmental challenges and under specific developmental stages (Cho and Paszkowski 2017). For example, TEs are reactivated in endosperm and pollen vegetative nuclei (Slotkin et al. 2009, Nagata et al. 2022). As these cells do not participate in fertilization, TE activation and mobilization in these cells, if any, does not result in any changes of the host genome sequence. This might indicate that active TEs in the non-germline cells may exert certain regulatory roles. Indeed, recent studies unveiled that RNA and DNA products of activated TEs act as potent regulators of gene expression. In this review, some of the most intriguing examples for biological functions of plant TEs will be highlighted.

## TE exaptation to transcriptional regulators

TEs encode for proteins that possess DNA-binding domains, for instance, integrase of LTR retrotransposons and transposase of DNA transposons. Some of these DNA-binding domains of TEs have been evolutionarily adopted to transcription factors and components of transcriptional super complexes. For example, the phytochrome A (phyA) signaling pathway of far-red light responses in *Arabidopsis* is mediated by FAR-RED ELONGATED HYPOCOTYLS 3 (FHY3) and FAR-RED-IMPAIRED RESPONSE 1 (Neff et al. 2000, Quail 2002). These proteins are domesticated Mutator-like transposases and regulate the phyA signaling by acting as transcription factors triggering the expression of downstream genes including *FHY1* and *FHY1-LIKE* (Lisch 2002, Hudson et al. 2003, Lin and Wang 2004). Furthermore, TEs can evolve into new protein components of large complexes that regulate gene expression. An example for this is ANTAGONIST OF LIKE HETEROCHROMATIN PROTEIN 1 (ALP1) in *Arabidopsis*, the sequence of which is derived from a Harbinger class TE. ALP1 is a component of the POLYCOMB REPRESSIVE COMPLEX 2 (PRC2), a protein complex that catalyzes the repressive histone mark H3K27me3 (Liang et al. 2015, Vijayanathan et al. 2022). Along with ALP2 that is also originated from a Harbinger transposon, ALP1 and ALP2 form variant PRC2 complexes that antagonize the deposition of H3K27me3 at the PRC2 target loci. ALP1 and ALP2 seem to suppress the incorporation of LIKE HETEROCHROMATIN 1, an essential accessory component of PRC2, thereby diminishing its H3K27me3 activity (Shaver et al. 2010, Mikulski et al. 2017, Velanis et al. 2020). These together imply that the DNA-binding ability of transposase proteins has been actively domesticated by the host to diversify protein complex functions (Biémont and Vieira 2006). More importantly, the extraordinary ability of TEs to rearrange their own DNA sequences enables generation of novel DNA and protein domains that can be recognized by and incorporated into the host super complexes, further diversifying their functions and regulation.

## TE-derived transcripts act as an miRNA target mimic

In the previous section, we showed examples that TE proteins can modulate the activity of a host protein complex by acting as a ‘fake’ component of transcriptional regulator complexes and interfering with the binding of essential elements. Now, we will introduce and discuss the recent discoveries of TEs in rice that impede the host gene regulators by acting as fraud RNA and DNA targets.

lncRNAs are cellular RNAs that are unable to encode for long peptides and usually act as architectural scaffold for various functions of proteins and RNAs. Studies in animals and plants have suggested that lncRNAs often originate from TEs (hereinafter referred to as TE-lncRNAs) (Kapusta et al. 2013). In plant genomes, many TEs are released from silencing at specific developmental stages, thereby facilitating the occurrence of tissue-specific TE-lncRNAs (Slotkin et al. 2009). In the rice genome, a TE-lncRNA named *MIKKI* is primarily expressed in root and epigenetically repressed in other tissues (Cho and Paszkowski 2017). Intriguingly, the *MIKKI* transcript contains an imperfect binding site for miR171 at its splice junction. It is important to note that the imperfect base-pairing between *MIKKI* and miR171 prevents the RNA cleavage of *MIKKI* transcripts but instead triggers the turnover of miR171. Such RNAs have been reported across eukaryotes and coined as miRNA target mimic, RNA decoy or competing endogenous RNA (Kartha and Subramanian 2014).

**Fig. 1:**
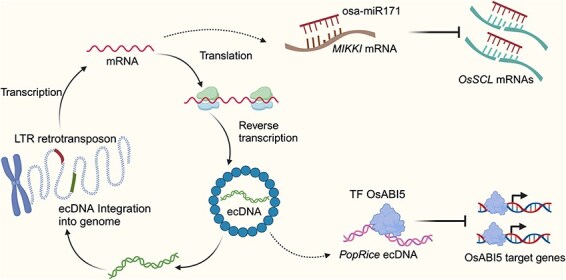
The life cycle of LTR retrotransposons and how they mimic endogenous biomolecules. *MIKKI* transcripts interact with osa-miR171, leading to its turnover and stabilization of *OsSCL* mRNAs. The GA-induced eccDNAs of *PopRice* compete with the ABA-responsive genes for OsABI5, facilitating the developmental transition from seeds to post-embryonic stage.

miR171 is a very well conserved plant-specific miRNA and targets transcripts encoding the SCARECROW-LIKE (SCL) transcription factors. In rice, *SCLs* are essential for root development and their ectopic expression leads to abnormal development of reproductive organs (Wang et al. 2010). On the other hand, rice miR171 is abundant in the reproductive tissues and scarce in rice roots. While the tissue-specific expression pattern of miR171 is conserved in both monocot and dicot plants, their underlying mechanisms are largely different. In dicot plants, the miR171 expression pattern is governed by the transcriptional control through the negative feedback loop of the miR171-SCL module (Ma et al. 2014), whereas in monocot plants the transcriptional activity of pri-miR171 is constant across tissues and the post-transcriptional control is critical for suppression of miR171 in the root (Cho and Paszkowski 2017). Indeed, the *MIR171* genes in rice lack the SCL-binding motifs in their promoters, and the suppression of miR171 in the root (and de-repression of *SCLs*) is mediated by *MIKKI*. It is also noteworthy that the generation of the *MIKKI* locus was dated to 1.2 million years ago, and its sequence is conserved only in the AA genome *Oryza* species (Cho and Paszkowski 2017). This implies that the *MIKKI* sequence is unique to the domesticated rice and suggests a possibility of the *MIKKI*-independent post-transcriptional control of miR171 in other monocot plants. In short, *MIKKI* serves as an intriguing example that rapid and frequent shuffling of DNA sequences within and among TEs can be exploited as an important source of lncRNAs, which can exert regulatory functions such as miRNA sequestration (**Fig. 1**).

## Extrachromosomal circular DNA as a functional biomolecule

Extrachromosomal circular DNA (eccDNA) is a closed circular DNA molecule and exists independently from chromosomal DNA (Hotta and Bassel 1965, Wu et al. 2019). The formation of eccDNA is mediated by diverse pathways, including DNA damage repair mechanisms such as the homologous recombination (HR) and non-homologous end joining (NHEJ) pathways (Meng et al. 2015), and the breakage–fusion–bridge cycle (Noer et al. 2022, Yang et al. 2022). TEs are the main source of eccDNAs because they possess repeat sequences that can be readily recognized by HR and NHEJ, and the linear ecDNAs of activated LTR retrotransposons are often circularized (Lanciano et al. 2017). Although their presence has been long known, the biological functions of eccDNAs have just started to be unveiled recently (Henriksen et al. 2022). For example, it was suggested that eccDNAs play important roles in the production of small RNAs (Paulsen et al. 2019) and the control of tumorigenesis (Kumar et al. 2017, Helmsauer et al. 2020, Kim et al. 2020, Koche et al. 2020).

In plants, the presence of eccDNAs was previously reported in wheat callus and tobacco leaves (Kinoshita et al. 1985). More recently, the advancement of next-generation sequencing expanded the catalog of plant eccDNAs significantly and revealed they are produced frequently from TEs (Roquis et al. 2021, Peng et al. 2022). LTR retrotransposons can produce eccDNA by circularizing the reverse transcribed linear ecDNA. In this process, the binding of integrase to the LTR region and its homodimerization are important because two DNA ends of linear ecDNA are brought to close proximity and likely recognized as a DNA double-strand break by the DNA repair machineries of the host (Fan et al. 2022). Although the roles of eccDNA in plants have not yet been fully elucidated, Peng *et al*. proposed an interesting hypothesis in their recent review, postulating that since TE-derived eccDNAs are actively produced upon global loss of DNA methylation and may produce small RNAs, TE-eccDNAs can act as a sensor of epigenetic silencing status that might signal to strengthen the small RNA-mediated genome defense (Peng et al. 2022).

## TE-derived eccDNAs act as a transcription factor sponge

Lanciano et al. was one of the first that systematically profiled eccDNAs in plants. In their ‘mobilome-seq’ pipeline, a *Copia*-type LTR retrotransposon was identified from the rice endosperm and named *PopRice* (Lanciano et al. 2017). *PopRice* belongs to the *Osr4* retrotransposon family but has undergone sequence divergence that conferred strong transcriptional activation in the endosperm. It is important to note that endosperm is a dead-end tissue, which does not carry over its genomic DNA to the progenies, and thus the activation of *PopRice* in the endosperm can possibly imply that the *PopRice* eccDNA might play certain regulatory roles to benefit the host plant. Indeed, inhibition of *PopRice* eccDNA biogenesis using tenofovir, a chemical reagent that prevents RNA-to-cDNA conversion, led to delay in rice seed germination (Brestovitsky et al. 2023, Chu et al. 2023). The expression levels of α-amylase, a hallmark gene of rice seed germination, were also reduced in the tenofovir-treated seeds, which is in line with the delayed seed germination phenotype (Chu et al. 2023). Importantly, the ABA treatment at a moderate level further suppressed the α-amylase expression, suggesting that the retardation of seed germination by tenofovir was due to the enhanced ABA sensitivity (Chu et al. 2023). Intriguingly, *PopRice* eccDNA contains several ABA-responsive cis-regulatory elements within its body region, while in the LTR region the endosperm-specific and GA-responsive sequence motifs are present. As expected, *PopRice* was strongly induced by exogenous GA treatment and hardly activated by ABA (Chu et al. 2023).

*PopRice* eccDNA was further tested for the interaction with rice ABSCISIC ACID-INSENTIVE 5 (OsABI5), an ABA-responsive transcription factor that is involved in seed germination, using the DNA affinity purification sequencing (DAP-seq) experiments (O’Malley et al. 2016, Chu et al. 2023). In agreement with the strong enrichment of ABA-related cis-regulatory motifs within *PopRice*, OsABI5 were able to bind to the internal region of *PopRice* (Chu et al. 2023). This proposes an interesting hypothesis that *PopRice* mediates the GA–ABA antagonism by acting as an OsABI5 sponge while being activated by GA. This idea was partly supported by a DAP-seq experiment with titration of the *PopRice* DNA in the interaction between OsABI5 and rice native genomic DNAs. Intriguingly, an excess provision of *PopRice* DNA weakened the affinity of OsABI5 to its DNA targets, indicating that *PopRice* can compete for ABA-signaling transcription factors with their authentic genomic targets (Chu et al. 2023). Overall, *PopRice* eccDNA acts specifically in the rice endosperm to restrict the action of ABA by sponging OsABI5 and thereby facilitate the transition to the post-embryonic development (**Fig. 1**; Chu et al. 2023).

## Conclusions and future prospects

In this mini review, we introduced several interesting cases of TE evolution that act as ‘fake’ targets of an miRNA and transcription factor, diminishing their action to ensure proper development in rice (**Fig. 1**). A similar notion was previously proposed in humans, suggesting that activated endogenous retrotransposons emulate viral infection, induce antiviral immune response, and thus can augment cancer immunotherapies, the process of which is known as ‘viral mimicry’ (Roulois et al. 2015, Chiappinelli et al. 2017, Chen et al. 2021). Such unique ability of TEs in mimicking cellular factors is attributed to their extraordinary feature of rapid sequence shuffling and transcriptional behavior responding to external and/or internal signals. For example, the miR171-binding sequence of *MIKKI* was generated from sequential integration of two retrotransposons that each provided splice donor and acceptor sites. In addition, both *MIKKI* and *PopRice* display tissue-specific gene expression patterns (in rice roots and endosperm, respectively), which allowed them to be integrated in the relevant gene regulatory networks that govern the developmental transition.

The investigation of TE-derived eccDNAs has emerged in recent years, largely due to advancements in the high-throughput sequencing methods like mobilome-seq. While these techniques offer opportunities to uncover previously unidentified TE intermediates in both plant and animal systems, a common limitation lies in the inability of effectively processing repetitive sequences. In the recent paper by Zhang et al., this constraint was overcome by leveraging long-read sequencing platforms, such as Oxford Nanopore sequencing. Through this approach, they unveiled complex structural variations of TE-eccDNAs in *Arabidopsis*, which was unable to be detected by the conventional short-read sequencing methods (Zhang et al. 2023). Moreover, long-read sequencing technologies will be particularly useful for non-model plant species with poorly annotated reference genomes as they allow for reference-free analysis of TE sequences. Therefore, future studies are encouraged to explore new eccDNAs across diverse plant species and elucidate their biological significance.

## Data Availability

No new datasets were generated or analyzed in this study.
